# Characteristics and Adverse Events in Acute Coronary Syndrome Patients with a History of Peripheral Arterial Disease

**DOI:** 10.5935/abc.20190177

**Published:** 2019-09

**Authors:** Iran Castro, Hugo Fontana Filho

**Affiliations:** 1 Fundação Universitária de Cardiologia - Instituto de Cardiologia (ICFUC), Porto Alegre, RS - Brazil

**Keywords:** Cardiovascular Diseases, Acute Coronary Syndrome, Peripheral Arterial Disease/complications, Diabetes Mellitus, Myocardal Infarction, Risk Factors

Cardiovascular diseases (CVD) are the leading cause of death in the world,^1^ with increasing numbers of cases in low and middle-income countries. In Brazil, it is estimated that 350,000 patients die each year due to CVD.^2^ The relationship between acute coronary syndrome (ACS) and peripheral atherosclerotic disease (PAD) is well established.^3,4^

Cross-sectional studies carried out in countries with genetic characteristics different from ours help us to evaluate the possible relationship between the characteristics of the patients evaluated and the increase in cardiovascular risk, and, despite their limitations, are good hypothesis generators.

The present study^5^ analyzed the characteristics of patients with ACS and PAD, showing that advanced age, diabetes, worse lipid profile and multiarterial disease were more prevalent in patients with ACS and PAD than in patients with ACS and without PAD. In addition, it suggested that patients with such an association have a worse prognosis.

Limitations of the present study were the facts of its retrospective analysis and the exclusion of patients at higher risk (prior acute myocardial infarction, acute myocardial infarction caused by thrombus detachment, intravascular surgery, patients with cardiogenic shock and post-cardiac arrest and gastrointestinal bleeding on admission), which could have shown an even higher risk in such patients, considering that generally patients with associated PAD have greater complications and worse prognosis. It is also worth noting that it was carried out in a single center.

However, the present study has great value for showing once again, as previous studies have demonstrated,^6^ the worsening of the prognosis of patients with ACS and PAD and which risk factors are most prevalent in this population, making possible the detection of subgroups of patients with ACS and PAD are more susceptible to a worse outcome and in this way emphasize their strict control. It is also necessary to offer the best treatment evidenced in the literature to such patients since real-life studies have shown that despite the higher risk of patients with ACS and PAD, they frequently receive less drugs with established benefit.^7^
